# Supplementary material for: Tissue-specific synergistic bio-priming of pepper by two Streptomyces species against Phytophthora capsici
Source: PLoS One. 2020 Mar 19;15(3):e0230531. doi: 10.1371/journal.pone.0230531 (PMC7082030; doi:10.1371/journal.pone.0230531)
Supplement: S1 Fig — IT20 (right) and SS14 (left) on ISP2 (A) and inorganic phosphate solubilizing medium after six days of incubation (B). IT20 shows inorganic phosphate solubilizing activity. These species have not inhibition interaction shows that the combination of IT20 and SS14 is compatible. (DOCX) [file pone.0230531.s001.docx]

**A**

**B**


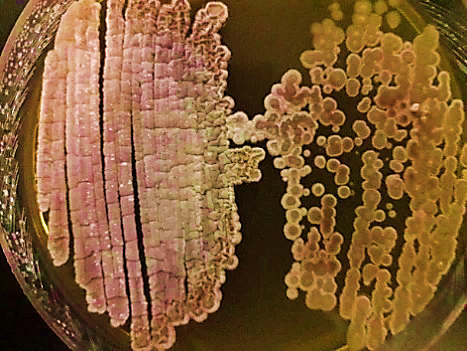

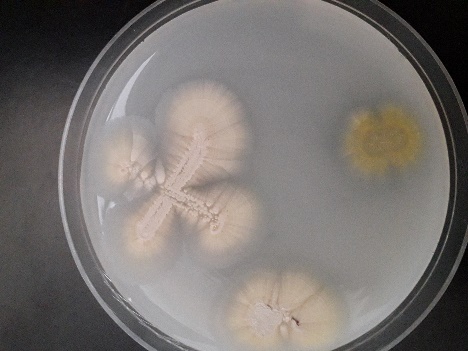


*IT20*

*IT20*

*SS14*

*SS14*

**S1 Fig.** IT20 (right) and SS14 (left) on ISP2 (**A**) and inorganic phosphate solubilizing medium after six days of incubation (**B**). IT20 shows inorganic phosphate solubilizing activity. These species have not inhibition interaction shows that the combination of IT20 and SS14 is compatible.


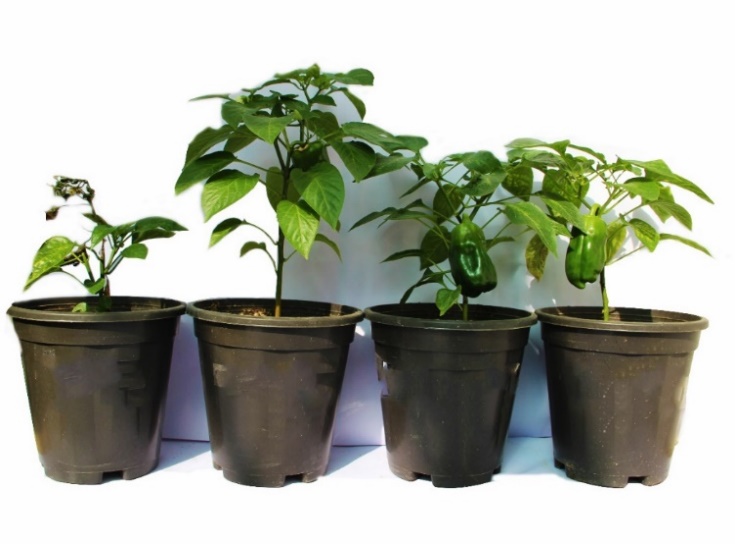


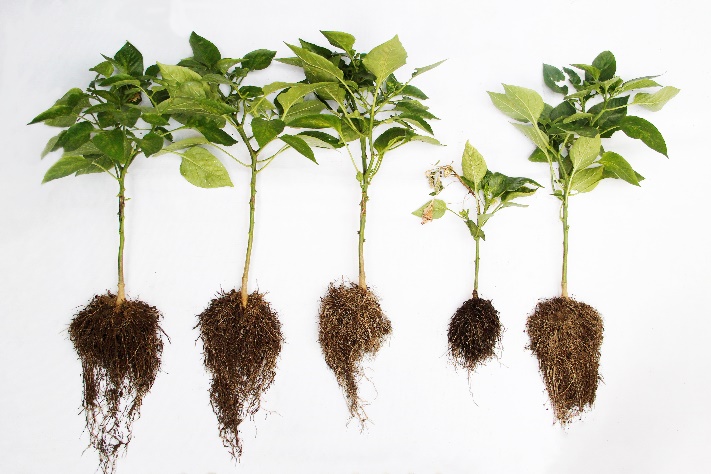


PC

C

PC

IT20+SS14+ PC

IT20+PC

SS14+PC

IT20+SS14 +PC

SS14+PC

IT20+PC

**S2 Fig.** Shoot and roots of pots inoculated with *Phytophthora capsici* after 80 days of bacterial treatments
