# Supplementary material for: Tissue-specific synergistic bio-priming of pepper by two Streptomyces species against Phytophthora capsici
Source: PLoS One. 2020 Mar 19;15(3):e0230531. doi: 10.1371/journal.pone.0230531 (PMC7082030; doi:10.1371/journal.pone.0230531)
Supplement: Attachment — Submitted filename: Response to Reviewers.docx [file pone.0230531.s003.docx]

Authors are grateful for reviewing the manuscript and good comments. We tried to improve and correct the manuscript according to the reviewer's comments. The answers (R) to the questions (Q) are as follow:

Q1. The manuscript needs major improvement in English language. It should re-written for clarity and accuracy of language.

**R.** Major improvements and changes were made in the manuscript, grammatical errors were corrected and conclusion was rewritten.

Q2. Name of the organisms must be in italicized throughout the text.

**R.** The name of the organisms italicized throughout the text.

Q3.The method section does not provide clear description of experimental procedure. This needs to be appropriately revised.

Q4. Please clarify how cfu/ml suspension of *Streptomyces* strains were achieved by using spores?

**R.** Method section was revised and clearly explained. We suppose all the questions are answered accurately. We cultivated one plug of seven days old bacterial strains in ISP2 media supplemented with 10 mM NaCl, then the spores were counted with hemocytometer.

Q5. Whether the *Streptomyces* strains were able to colonize the root, leaf or fruit of the plant following treatment with the culture?

**R.** Indeed, we only noted the effect of soil treatment by these two species and reported that *Streptomyces* were able to colonize the root plant following re-isolation and culturing. We did not examine leaf and fruit colonization.

Q6. What was the level of expression of plant defense genes during the 7-days period following treatment of the plant with the *Streptomyces* spp. but before inoculation of the *P. capsici* into the plant?

**R.** Just, soil drench with chemical inducer BABA (10mM) was performed 48 h before *P.capsici* inoculation. After seven days of bacterial treatment (to establish), plants were inoculated with the pathogen. The first disease symptoms were observed on pepper plants seven days after pathogen inoculation. So, after seven days of *P.capsici* inoculation leaves and fruits (by length 1 cm) were harvested for study. We have two groups of treatments **inoculated** and **non-inoculated** with pathogen.

Q7. Table 1 seems unnecessary and should be removed. Necessary information may be included in the materials and methods.

**R.** Table 1 was removed and included in the content of material and methods anywhere needed.

Q8. There is no description of Fig. 1 in the text??
Q9. Superscripts in Table 3 should be explained in note under the table.

**R.** subscription into note and description of Fig 1 were done.

Q10. What was the rational for measuring shoot length 30 days after inoculation and plant weight on 80 dai? Whether these measurements were done only once during the entire period of experiment?

All of them were conducted during 30-80 days after treatment in two time repeats of experiments. We observed difference in plant height promotion after 30 days of treatment. At the end of study we observed some treatments have less reproductive promotion but more vegetative growth promotion, so we measured plant weight.

Q11. Results pertaining to *Phytophthora* fruit blight assay of treatments, contained in Fig. 6 should be moved at the end of the result section after description of Fig 5.

**R.** The results of fruit rot assay was moved after Fig 5.

Q12. Since several *Streptomyces* spp. are reported to induce systemic acquired resistance in plants and protect them against phyto-pathogenic fungi, what is novel in this study?

The results showed that phosphate solubilizing *Streptomyces* strain has the potential to induce systemic resistance and reduce the effects of leaf blight disease in compatible combinations in the greenhouse but expression analysis in two different tissues was distinct. Such findings are important, as they open doors to study the mechanisms and the interactions between the genetic background and priming in different tissues of the host plant.
