# Supplementary material for: Tissue-specific synergistic bio-priming of pepper by two Streptomyces species against Phytophthora capsici
Source: PLoS One. 2020 Mar 19;15(3):e0230531. doi: 10.1371/journal.pone.0230531 (PMC7082030; doi:10.1371/journal.pone.0230531)
Supplement: Attachment — Submitted filename: Response to Reviewers R2.docx [file pone.0230531.s004.docx]

Dear Marie-Joelle Virolle,

I am grateful for reviewing the manuscript and good comments. We tried to improve and correct the manuscript according to the reviewer's comments and the revised manuscript is submitted.

C2. The manuscript needs major improvement in English language. It should re-written for clarity and accuracy of language.

**R.** Major improvements and changes were made in the manuscript, grammatical errors were corrected and conclusion was rewritten.

Best regards,

Naser Safaie
